# Influences of community engagement and health system strengthening for cholera control in cholera reporting countries

**DOI:** 10.1136/bmjgh-2023-013788

**Published:** 2023-12-06

**Authors:** Stephanie Ayres Baličević, Kelly Osezele Elimian, Carina King, Karin Diaconu, Oluwatosin Wuraola Akande, Vivianne Ihekweazu, Hanna Trolle, Giulia Gaudenzi, Birger Forsberg, Tobias Alfven

**Affiliations:** 1 Department of Global Public Health, Karolinska Institutet, Stockholm, Sweden; 2 Exhale Health Foundation, Abuja, Nigeria; 3 Institute of Global Health, Queen Margaret University, Edinburgh, UK; 4 Department of Epidemiology and Community Health, University of Ilorin Teaching Hospital, Ilorin, Nigeria; 5 Office of the Managing Director, Nigeria Health Watch, Abuja, Nigeria; 6 Protein Science, SciLifeLab, Stockholm, Sweden; 7 Sachs’ Children and Youth Hospital, Stockholm, Sweden

**Keywords:** Cholera, Health systems, Public Health, Review, Medical microbiology

## Abstract

The 2030 Global Task Force on Cholera Control Roadmap hinges on strengthening the implementation of multistranded cholera interventions, including community engagement and health system strengthening. However, a composite picture of specific facilitators and barriers for these interventions and any overlapping factors existing between the two, is lacking. Therefore, this study aims to address this shortcoming, focusing on cholera-reporting countries, which are disproportionately affected by cholera and may be cholera endemic. A scoping methodology was chosen to allow for iterative mapping, synthesis of the available research and to pinpoint research activity for global and local cholera policy-makers and shareholders. Using the Arksey and O’Malley framework for scoping reviews, we searched PubMed, Web of Science and CINAHL. Inclusion criteria included publication in English between 1990 and 2021 and cholera as the primary document focus in an epidemic or endemic setting. Data charting was completed through narrative descriptive and thematic analysis. Forty-four documents were included, with half relating to sub-Saharan African countries, 68% (30/44) to cholera endemic settings and 21% (9/44) to insecure settings. We identified four themes of facilitators and barriers to health systems strengthening: health system cooperation and agreement with external actors; maintaining functional capacity in the face of change; good governance, focused political will and sociopolitical influences on the cholera response and insecurity and targeted destruction. Community engagement had two themes: trust building in the health system and growing social cohesion. Insecurity and the community; cooperation and agreement; and sociopolitical influences on trust building were themes of factors acting at the interface between community engagement and health system. Given the decisive role of the community–health system interface for both sustained health system strengthening and community engagement, there is a need to advocate for conflict resolution, trust building and good governance for long-term cholera prevention and control in cholera reporting countries.

**WHAT IS ALREADY KNOWN ON THIS TOPIC**

- Although cholera remains a persistent threat to health security in many cholera reporting countries, there is no known previous synthesised evidence as to specific facilitators and barriers to implementing health systems strengthening and community engagement interventions, and how these relate to the community–health system interface, for actualising the Global Roadmap Strategic Goals—decrease cholera deaths by 90% and eradicate cholera in about half of cholera endemic countries by 2030.

**WHAT THIS STUDY ADDS**

- Global solidarity and international pressure to end armed conflicts would strengthen not only healthcare systems, community engagement and service delivery at the community–health system interface, but would also benefit cholera control in settings made fragile by armed conflicts or civil unrest.
- Boosting awareness of power dynamics in managerial positions, through the development of governance skills in front-line workers, can promote cooperation between health system actors and has the potential to regulate and positively influence community–health system interface power imbalances.
- Cholera messaging that inappropriately associates cholera transmission routes (such as faecal-oral transmission routes) to individual attributes (such as personal hygiene) should be avoided, as this can lead to individual and community shame and marginalisation.

**HOW THIS STUDY MIGHT AFFECT RESEARCH, PRACTICE OR POLICY**

- While focusing on the community–health system interface would be crucial in sustaining strengthening of both healthcare systems and community engagement for cholera control in cholera reporting countries, targeting cholera ‘hotspots’ without considering existing power dynamics and community trust issues may risk increasing distrust in government institutions or marginalising fragile communities.

## Introduction

Cholera, characterised by profuse watery diarrhoea, remains a significant threat to global health security. Cholera is contracted through ingestion of water or food contaminated with the bacterium *Vibrio cholerae*. Globally, cholera is a disease of inequity, occurring most in conditions of poverty with poor basic water, sanitation and hygiene (WASH) services, including settings such as periurban slums and camps for internally displaced persons.^1^Fragile settings (countries or settings with socioeconomic, environmental or security volatility, with poorly performing or disintegrating public health services, and with poor community resources^2 3^) afflicted by humanitarian disasters—man-made or natural—or flooding due to climate change, continue to experience new cholera epidemics.^1 4^ Two such settings, Nigeria and Yemen, still have persistent unsafe water sources^5 6^ and have recently experienced cholera epidemics.^7 8^ New outbreaks have occurred in Syria, where conflict-damaged water infrastructure leads to unsafe water sources. Similarly, Haiti has seen cholera remerge, as water treatment facilities are deprived of fuel by armed gangs.^9^ At least 47 countries are still cholera endemic,^1^ with about 2.86 million infections, and 21 000–143 000 deaths occurring annually.^10^ Notably, 60% of cholera cases occur in sub-Saharan Africa in certain ‘hotspots’ (specific geographic areas with persistently high cholera burden), where 40– 80 million people live.^1^ Cholera, endemic in the WHO Africa region, is still contributing to cumulative cases of 211 643 and cumulative deaths of 3953 as of July 2023.^11^ To avoid classifying countries based solely on income levels and to assist in highlighting commonalities between settings,^12^ the imperfect term ‘cholera reporting countries’ will be employed throughout this study to designate countries or settings within countries as being disproportionately affected by cholera. Given the ongoing cholera burden, the Global Roadmap to 2030 of the WHO Global Task Force on Cholera Control (GTFCC) outlines its aims to decrease cholera deaths by 90% and eradicate cholera in 20 of 47 endemic countries by 2030.^1^ Three priority areas include early detection and control of outbreaks, targeted multisectoral interventions in cholera hotspots and international collaboration for technical support and partnership.^1^ The GTFCC’s roadmap highlights health system strengthening (HSS) interventions for cholera control in both hotspot and outbreak settings. For sustainable cholera control, the GTFCC Roadmap calls for community engagement (the dynamic process of relationship building between communities and health system stakeholders to collectively improve health outcomes^13^) to promote hygiene, behavioural change around sanitation (especially open defecation) and sustained WASH resource management.^1 14^

Fragile settings and fragile populations (populations with security, poverty, inherent vulnerability or marginalisation concerns)^2 3 15^ are most at risk of cholera.^1^ Additionally, fragile settings are less likely to be able to deal with acute shocks such as cholera epidemics. Also, fragile populations frequently display issues with trust between HS actors and communities.

Th community–health system relationship, also described as the community–health system interface, indicates a health system’s resilience.^2 15^ Additionally, trust has been reported as a ‘critical determinant of health seeking’^3^ and playing a crucial role in promoting active participation and adherence to recommendations and uptake of public health interventions, especially during emergencies.^16^ Community engagement and community empowerment are generally believed to assist in re-establishing trust and balancing unequal power dynamics at the community–health system interface. There is a lack of composite literature on how to achieve community engagement and empowerment in fragile settings and populations, or at community–health system interfaces characterised as fragile.^3^

Recent global cholera vaccine stockpile shortages have highlighted the need for WASH improvements to provide long-term solutions to cholera. WASH improvements need political and community engagement and support.^17^ In addition, effective health system functioning appears to be influenced by community engagement.^2 3 15^ Therefore, exploring definite factors influencing community engagement and HSS for cholera control, and further to synthesise available evidence to suggest refinement of efficient cholera control interventions, will be a worthwhile endeavour. Concurrently, as the community–health system interface in cholera control appears to be minimally investigated, this also needs to be examined. Hence, we aimed to identify and describe the specific facilitators and barriers that affect sustained community engagement and HSS for cholera control in cholera reporting countries, and examine how these overlap, with a view to contributing to achieving the GTFCC’s goals

## Methods

Due to the complexity of the identified under researched concepts of community engagement, HSS and the community–health system interface around cholera prevention and control, we conducted a scoping review. Scoping reviews allow for a necessary broad perspective on the research questions, inclusion of all forms of research material, as well as mapping, outlining synthesised evidence and identification of research gaps for propagation to relevant stakeholders. The five-stage Arksey and O’Malley framework,^18^ and modifications applied by Levac *et al*,^19^ were used to guide the research.

### Stage 1: identifying the research question

The research questions were: (1) What are the facilitators and barriers and their mechanisms that influence sustained healthcare system strengthening in cholera control in cholera reporting countries? (2) What are the facilitators and barriers and their mechanisms that influence community engagement in the control of cholera in cholera reporting countries? (3) What factors are operating at the interface between community engagement and healthcare system strengthening for cholera control in cholera reporting countries? Definitions of the terms used in the research questions are described in table 1.

**Table 1:** Definitions of research question terms

Community engagement	Creation and outcome of relationship building between communities—distinct groups of people who share some commonality—and health system stakeholders, facilitating better health; recognised as either community involvement, participation, mobilisation or empowerment, depending on the level of community involvement in said process.^13^
Health system strengthening	Interventions to improve any functions of, or facilitate interactions between, the WHO Health Systems building blocks (service delivery; health workforce; information systems; medical products, vaccines and technologies; financing; governance and leadership), resulting in improved and sustained health outcomes and equitable service delivery.^15 56^
Community–health system interface	The confluence of community processes and public health provision is characterised by dynamic interactions between the two, and is characterised by service delivery quality and coverage.^2 3^

### Stage 2: identifying relevant documents

An iterative process was followed, aided by Karolinska Institute librarians, to conduct a broad search of three electronic databases—PubMed, CINAHL and Web of Science. The data source determined the search strategy; details are available in online supplemental file 1. Search terms related to cholera, community engagement and health system components were derived from the definitions described in table 1. The search time frame was restricted to 1990–2021. Documents were restricted to those published in English Language. Search results were uploaded to Rayyan. This stage in the research was started on 10 December 2019 and was completed on 24 February 2021.

10.1136/bmjgh-2023-013788.supp1 Supplementary data

### Stage 3: selecting relevant documents

The selection of relevant documents was performed by SA, with support from KOE, as needed. Selection criteria were iteratively derived through repeated selection rounds and reapplied to selected documents when altered; details are in table 2. Documents with primary document setting designated as being in a high income country (based on income level groups assigned by the World Bank^20 21^) were excluded, as none of these settings fell within the description of cholera reporting country employed in this study. Further selection rounds focused on identifying explicitly reasoned health system and community functioning facilitators, barriers and mechanisms. Documents primarily relating to knowledge, attitudes and practice around cholera and/or WASH, were excluded in the final selection rounds due to analysis time constraints. Uncertainties around inclusion were resolved through discussion between SA and KOE.

**Table 2:** Inclusion and exclusion criteria applied to the selection process

Inclusion criteria	Exclusion criteria
Peer-reviewed journal article, book chapter, WHO publication, scientific journal news article	Editorial, letter, commentary, advice, opinion piece, book review, historical article
Document includes statement(s) relating to cholera prevention or control; statement(s) relates to: Functioning and interactions of health systems and their building blocks^56^ and reasoning stated. Communities and their (in)ability to engage (inform, consult, involve, collaborate, empower)^13^ and reasoning stated. Interaction(s) between communities and health systems and reasons for form of interaction stated.	Prediction and modelling studies
	Individual knowledge, attitudes and practice focus (if not explicitly discussing health systems or communities functioning)
	WASH interventions focus (if not explicitly discussing health systems or communities functioning)
Current cholera document focus (outbreak, prevention campaign, retrospective review), in endemic or epidemic setting and after 1990	Multiple disease focus (diarrhoea; infectious disease burden)
	Global cholera control focus (global surveillance reporting; global vaccine regulation)
*Vibrio cholera* O1 and O139	non O1 or O139 vibrio species
Document in English	Document not in English
	Documents where full text not available electronically
Primary document setting being lower-middle- income countrie, as per the World Bank classification^20 21^	Primary document setting being higher income country, as per the World Bank classification.^20 21^

### Stage 4: data charting

This stage describes the synthesis and interpretation of information from selected documents. Using MS Excel, a data-charting form was developed based on the data extraction table used by Diaconu *et al*.^3^ Data extraction entailed summarising and recording pertinent text passages in the MS Excel spreadsheet. An independent, iterative data charting process, employing a descriptive-analytical method was conducted to extract the results^19^ (a cross-section of the data extraction form is seen in online supplemental file 2).

10.1136/bmjgh-2023-013788.supp2 Supplementary data

### Stage 5: collating, summarising and reporting the results

Results were derived in two ways: first, a bibliometric analysis, using Stata V.16 (StataCorp), collected general document information which was presented using descriptive statistics; second, an inductive version of Braun and Clarke’s approach to thematic analysis was followed to identify themes in the collated dataset.^22^ Details of the coding process may be seen in online supplemental file 3. This scoping review was reported per the PRISMA-ScR checklist.^23^

10.1136/bmjgh-2023-013788.supp3 Supplementary data

### Patient and public involvement statement

Patients or the public were not involved in the conduct of this study given its design and methodology.

## Results

**Figure 1:**
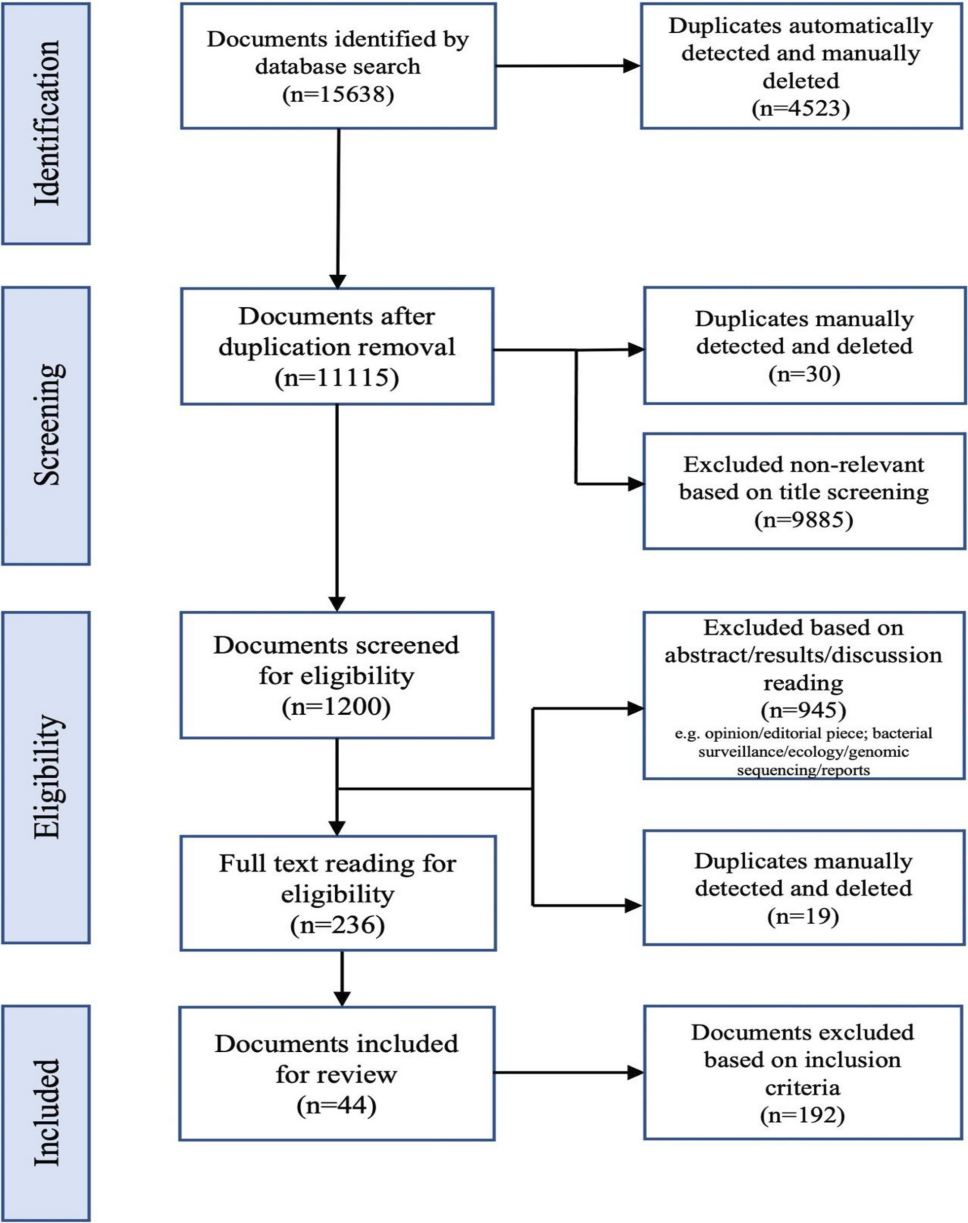
A flow chart showing the identification and screening process for selecting the study documents per the PRISMA-ScR.

The search of the three databases identified 15 638 documents (online supplemental file 4), of which 44 were included in this review (see figure 1).

10.1136/bmjgh-2023-013788.supp4 Supplementary data

### Descriptive analysis

The characteristics of included documents are summarised in table 3. Documents were published between 1995 and 2020, with the majority (75%) in the most recent period of 2011–2020. A wide range of countries were represented, with the most (18%) in Haiti. However, half of the documents are from sub-Saharan African countries, particularly Zimbabwe (n=6, 14%). Most documents referred to national level or multiple regions, while slightly more urban (27%) than rural (21%) settings were described. Furthermore, endemic cholera settings were more common (23%), and 21% of documents were in the context of armed conflict or civil unrest. There was a wide range of study designs, with 32% using mixed methods.

**Table 3:** Baseline characteristics of included documents (N=44)

Characteristic	Frequency	%
Document identifiers		
Author’s affiliation		
Academic	20	45
Government	13	30
Government/academic	1	2
International NGO*	4	9
Local NGO	1	2
Multinational NGO	3	7
News†	2	5
Publication period		
1991–2000	2	5
2001–2010	9	20
2011–2020	33	75
Document description		
Country		
Brazil	1	2.3
Democratic Republic of Congo	3	6.9
Dominican Republic	1	2.3
Ethiopia	1	2.3
Ghana	1	2.3
Haiti	8	18.2
Kenya	3	6.8
Madagascar	1	2.3
Mexico	1	2.3
Mozambique	1	2.3
Nigeria	4	9.1
Papua New Guinea	1	2.3
Peru	1	2.3
Senegal	1	2.3
Swaziland	1	2.3
Tanzania	1	2.3
Uganda	3	6.8
United Republic of Tanzania	1	2.3
Yemen	3	6.8
Zambia	1	2.3
Zimbabwe	6	13.6
Country setting		
Multiple Regions‡	5	11
National	14	32
Region§	4	9
Rural¶; multiple regions	2	5
Rural; region	7	16
Urban setting**	12	27
Context regarding cholera status		
Endemic††	10	22.7
Epidemic	14	31.8
Epidemic and endemic	20	45.4
Context situational		
Armed conflict	6	13.6
Civil unrest	3	6.8
Earthquake	2	45.4
Unspecified/NA	33	75
Document methodology		
Study design		
Case–control	3	6.8
Cross-sectional	2	4.5
Mixed methods	14	31.8
Narrative review	5	11.3
News	4	9
Qualitative case study	4	9
Qualitative case study and review	2	4.5
Quasi experimental	5	11.3
Review	5	11.3

*NGO: Non-governmental organisation.

†News item in scientific journal.

‡More than one region described.

§Governmentally designated large administrative area.

¶Setting described as rural.

**Setting described as urban.

††Confirmed cholera case within last 3 years and local transmission evidence.^1^

### Thematic analysis

In this review, we identified 10 facilitators and 9 barriers pertaining to 6 themes relating to sustained cholera control for HSS and community engagement. Three themes delineating factors affecting the community–health system interface were ascertained. Table 4 outlines the facilitators and barriers grouped into themes. Following, we explain the themes identified respectively and highlight some facilitators and barriers we identified as particularly important.

**Table 4:** Themes, facilitators, barriers and mechanisms relating to health system strengthening, community engagement and community–health system interface

	Theme	Facilitator	Barrier
Health system strengthening	1. Insecurity and targeted destruction		Insecurity disrupts or delays health system functioning and strengthening efforts, due to: Decreased HCW numbers (due to migration/death/fear/restricted workplace access)^24 25^ Information system delays (surveillance/supervisory feedback)^26^ Medical technologies (supplies) distribution disruption^27 28^ External aid provision difficulties (HCWs/expertise, medical supplies)^25 27^ Service delivery restrictions due to destruction of health facilities and infrastructure support lines^24 25 27–29^
Health system strengthening	2. Health system cooperation and agreement with external actors	Cooperation and agreement promote information sharing,^33 34 37^ especially when: Multiple health providers involved (eg, OCV campaign^37 43^/surveillance programme improvements^38 85^) Clear role designation respected^27 29 33^ Information regularly shared^33^ Sustained collaboration encourages adoption of HS changes^35^	Poor cooperation and disagreement delay cholera response coordination^27 29 33 34^
Health system strengthening	3. Maintaining functional capacity in the face of change	User friendly technological tools (coded mobile phone/tablet device) enable task fulfilment, during Data collection in OCV campaign/surveillance^43 85 86^ Systems and HCWs that are context adapted and flexible to changes facilitate functioning and HSS,^35 43 86^ especially when: Flexible and adequate financing available^27 33 38^ Adaptable information systems used (data collection integration in OCV campaigns^33 37 38 43^) Flexible work practices of HCWs (HCWs creating contextual contingency plans when needed^35 37 38^) Adequate numbers of HCWs and essential trained staff enable sustained HS functioning^37–39^	Lack of flexibility and adaptation obstruct ongoing functional capacity of HS interventions, through: Inappropriate tools (OCV cold chain requirements)^43^ Lack of contingency plans when challenges arise (information/medical technologies)^26 29 33 36^ Inadequate vital components hamper capacity of HCWs/facilities to sustain function, especially regarding: Laboratory capacity (missing essential trained staff/supplies/equipment)^26 38 42 87^ Health service delivery (inadequate HCW numbers/insecurity)^25 27 28 32 88 89^ Health workforce, through poor performance due to: system factors (inadequate supplies/HCWs/workload)^26 33 36 38 87 90^ inadequate individual capacity/knowledge/skills/experience^42 90–93^
Health system strengthening	4. Good governance, focused political will and sociopolitical influences on the cholera response	Strong leadership and proactive political will facilitate HS functioning Dedicated supervision and feedback are key in HCW training and capacity building,^35^ and also bolster HCW motivation and raise awareness of critical cholera-related tasks^85^	Poor supervisory feedback impedes efficient supply chain management and cholera response capacity,^27 36 42^ by: Limiting HCW attitude and practice improvements^26^ Creating HCW frustration and poor working relationships between staff and managers^36^ Weak leadership and poor coordination limit resource management and appropriate cholera response actions,^27^ through: Ineffective human resource management^33^ Curbing intergovernmental and financial mobilisation^32 34^ Socioeconomic influences and politicisation of cholera responses negatively influence governance functioning, through: Politically instigated interfering policy or legislation preventing external aid provision^44 88^ Denial or delayed acknowledgement of a cholera outbreak or its severity, due to: Fear of negative economic repercussions (in areas dependant on tourism revenues)^41 44^ Politicisation of the response^54 55 88 94^ Insecurity or civil war due to political insecurity^24 25 27 28 30^
Community engagement	5. Building trust in the health system	Community representatives are crucial to engaging communities and building trust in HS interventions,^29 31 37 41 44–47 50 52^ and described as: Being of various forms (religious leaders/tribal, traditional or village leaders/appointed community leaders/teachers/trusted individuals)^29 31 37 41 44 45 47^ Providing mentorship/encouragement for behavioural change (latrine building)^47^	Inappropriate language and communication strategies breed suspicion and mistrust of HS interventions, if: Uncontextualised language/names used (eg, names of water purification tablets) Negative local connotations/interpretations not accounted for^29 31 48^
Community engagement	6. Growing social cohesion	Collective productions of music and dance grow community interest in HSS interventions Existing community organisations can foster development of community mobilisation and empowerment, for example, Civil society organisations^37 43^ Community movements (eg, gwoupman peyizan)^46^	Cholera stigma blocks individual and community participation, acting: Individually as a barrier to health seeking and reporting^32 40 48^ collectively by: Preventing assistance to sick community members Supressing reporting^40^ Reinforcing pre-existing stigmatisations (eg, marginalised groups)^78^
Community–health system interface	7. Insecurity and the community		Insecure situations prevent growing community awareness by: Preventing access to treatment for community members (out of fear of injury/death due to bombing during usage)^30^ Preventing hygiene promotion efforts by HCWs (through location specific access restrictions)^27^ States of insecurity restrict service delivery capacity, through: Lack of cholera response interventions coverage in affected areas^25 27^ Limited facility opening times in insecure areas^24 29^
Community–health system interface	8. Cooperation and agreement	Cooperation between HS actors, community representatives and trained community members promotes successful engagement and HSS interventions,^33 36 37 41 43 52 53 95^ especially: For feedback to tailor hygiene messaging language^43 53 95^ Recruitment of local volunteers^37^ Community development^46^ Contextualised community surveillance Cholera prevention and control measures (eg, water chlorination)^34 41 53^	Lack of cooperation and agreement between HS providers causes confusion and mistrust in communities
Community–health system interface	9. Sociopolitical influences on trust building		Political dissonance and interference harms trust in the community–HS relationship,^31 32 40 48 54 94^ notably: Preventing community-level officials from engaging with communities^32^ Restricting service delivery^42 44 54 55 88 94^

HCW, healthcare worker; HS, health system; HSS, health system strengthening; OCV, oral cholera vaccine.

### Health system strengthening

#### Theme 1: insecurity and targeted destruction

States of insecurity, such as civil war or community unrest, and associated looting or destruction of facilities, describe the setting for this barrier.

##### Barrier: insecurity disrupts or delays HS functioning or strengthening efforts

This barrier acts across multiple health system blocks (see table 4).^24–31^ Notably, delays or restrictions on external aid impede strengthening efforts.^26 32^ This was particularly evident in Yemen from 2014 until now, where targeted bombing of health facilities and water infrastructure, teamed with blockades of supply chains and restrictions on humanitarian aid access, acted as barriers to health service delivery.^25 27^

#### Theme 2: HS cooperation and agreement with external actors determine sustained HSs interventions

This theme’s facilitators and barriers concern collaboration between governmental and non-governmental stakeholders, such as national government actors working alongside local or international non-governmental organisations (NGOs).

##### Facilitator: cooperation and agreement promote information sharing

Cooperation is vital in multisectoral outbreak responses, where daily collaborative meetings are noted as a mechanism to improve information flow and sharing, thereby improving response coordination.^29^ The opposite acts as a barrier,^27 29 33 34^ which can lead to delayed construction of initial treatment centres and delayed adequate cholera response capacity development.^29^

##### Facilitator: sustained collaboration encourages the adoption of HS changes

Trust, enabling sustained HS strengthening interventions, is built through continuous interactions between external providers and local health system actors; one example was the implementation of a digital pharmacy inventory to improve medical supply chains in Haiti in 2012.^35^

#### Theme 3: maintaining functional capacity in the face of challenges

This theme encompassed facilitators and barriers affecting the capability of HS block components to continue functioning. Barriers were particularly notable.

##### Barrier: lack of flexibility and adaptation obstructs the ongoing functional capacity of HS interventions

Lack of flexibility and adaptation to change, such as creating tailored contingency plans when needed,^29 33 36^ prevents the ability of an HS to continue functioning. An example is the disruption caused by a ban on motorcycle use due to the armed insurgency in Northeast Nigeria in 2017: given that surveillance reports were transported by motorcycle and no contingency plans were in place, the lack of this transport method severely retarded surveillance report collection and information system functioning.^26^

##### Barrier: inadequate vital components inhibit the capacity of healthcare workers/facilities to sustain functioning

Unfilled essential staff posts such as managerial or critical staff positions are among the barriers to difficulties in multiple HS blocks (see table 4). In Papua New Guinea during its 2009 cholera epidemic; inadequate essential staff, in the form of trained field epidemiologists and data managers, resulted in insufficient data management processes and poor coordination between the country’s provinces, hampering the success of HSS interventions.^33^

#### Theme 4: good governance, focused political will and sociopolitical influences on the cholera response

Facilitators and barriers in this theme describe the effects of governance measures, such as engaged leadership, political will and dedicated supervision and feedback, on the success of HSS interventions. The barrier created by socioeconomic and political influences is also included due to its effects on governance functioning (see table 4).

##### Facilitator: strong leadership and proactive political will facilitate HS functioning

Capacity building of all HS blocks is boosted by dedicated leadership and engaged political will.^29 33 37–40^ At the local level, strong leadership demonstrated commitment to an intervention, especially in the face of possible civil unrest and resistance to interventions.^37^ Strong leadership promoted pre-emptive supply and resource coordination at a regional or state level, even when national leadership was unsupportive.^41^ Nationally, an example of strong leadership with focused political will was seen in Mexico in 1991, with the declaration of cholera as a national security issue resulting in prioritised coordination across ministries and agencies.^39^

##### Facilitator: dedicated supervision and feedback are critical in HCW training and capacity building

Training methods that included practical demonstration and in-person collaboration with supervisors or experienced peers,^35^ assisted in familiarising healthcare workers (HCWs) with the disease and helped to combat the fear of the disease in cholera non-endemic areas.^33 42^

### Community engagement

#### Theme 5: building trust in the health system

The facilitator and barrier in this theme relate to how trust between communities and HS actors or in interventions is promoted or negated (see table 4 for details).

##### Facilitator: community representatives are crucial to engaging with communities and building trust in HS interventions

As community members themselves, community representatives appropriately contextualised information for communities. They enabled cooperation, prevented misunderstandings and helped to dispel false beliefs,^31 37 43^ which promoted information dissemination around cholera control and prevention efforts,^37 44–46^ especially for interventions such as oral cholera vaccines (OCV).^37 43^ Notably, trust was facilitated through physical demonstration of possible contentious interventions by trusted community representatives, such as publicly taking medications, water or OCV.^29 31 45 47^

##### Barrier: inappropriate language and communication strategies breed suspicion and mistrust of HS interventions

Cholera messaging around hygiene promotion, when focused on individual behaviours or qualities as determinants of infection risk, potentially induced feelings of persecution,^48^ blame or shame in individuals or communities.^32 49^ This was particularly noted when cholera-causative beliefs were not the same as those presented by authorities.^31^ This was evident in South and Central America, where messaging appeared to make moral judgements of certain individuals or communities through metaphorical associations with social, economic status or personal hygiene.^32 48 49^

#### Theme 6: growing social cohesion

This theme concerns the facilitators and barriers that affect communities’ ability to work collectively, allowing for community engagement (see table 4 for details).

##### Facilitator: collective productions of music and dance grow community interest in HSs interventions

Culturally appropriate music and dance involving community representatives could foster community enthusiasm and interest in hygiene promotion events^50^ and bring a sense of community pride and cohesion to particular communities.^51^

##### Barrier: cholera stigma blocks individual and collective participation

When collective stigma is systematised, through legislation or policies that appear to target certain groups, stigmatised communities may be disenfranchised. This can lead to stigmatised communities being unable to participate in a collective society, as occurred through legislation around migration documentation of Haitians in the Dominican Republic in 2012.^49^

### Interface between the health system and communities

#### Theme 7: insecurity and the community

This theme outlines factors that arise in insecure states or situations and how these act at the community–health system interface. Table 4 details how the growth of community awareness was prevented, and service delivery restrained. Notably, politically motivated resistance to cholera preventive efforts within communities may lead to violence towards persons associated with cholera prevention and control efforts, especially when the cause of a cholera outbreak is disputed. For example, this resistance has led to attacks on HCWs and deaths in Mozambique in 2009.^31^

#### Theme 8: cooperation and agreement

Cooperation relates to the cooperation of health system actors, such as HCWs, or community members trained in cholera prevention and control interventions, with other community members. Details of factors identified can be found in table 4. Cooperation between HS providers and community representatives was beneficial in areas of potential resistance to HS interventions or insecurity.^31 37 43 52^ A lack of cooperation and cohesion between HS providers when enacting HSS interventions, caused confusion and mistrust among patients or community members.^29 32 47 53^

#### Theme 9: sociopolitical influences on trust building

Political influences on trust in the community–HS relationship are outlined in table 4. Social factors such as the long-term lack of public services, especially when perceived as politicised, appeared to influence trust in the government’s ability and willingness to engage with communities.^31 32 48 49 54^ This could progress to a community’s sense of being politically abandoned and disenfranchised, obstructing participation^54 55^ or actively resisting cholera control and prevention efforts.^31 48^

## Discussion

We identified three themes influencing HSS for cholera control in cholera reporting countries: health system cooperation and agreement with external actors; maintaining functional capacity in the face of change, and good governance, focused political will, and sociopolitical influences on the cholera response. The theme of insecurity and targeted destruction featured a notable barrier. Two themes were described for community engagement: building trust in the health system and growing social cohesion. Three themes of factors acting at the community–health system interface were found, namely: insecurity and the community; cooperation and agreement; and sociopolitical influences on trust building. Multiple facilitators and barriers were identified for most themes.

### Governance and the community

The predominance of governance measures identified throughout three of the four themes for HSS emphasise the importance of good governance for cholera control and response. Although theme 4concerned governance measures directly, theme 2 and theme 3 also covered facilitators and barriers related to governance measures, such as cooperation, collaboration and coalition building, along with leadership and management (eg, human and material resources); all are implicit in good governance.^56^ Hence, while the GTFCC Roadmap highlights leadership and coordination as a pillar in the 2030 cholera response plan,^3^ good governance should be added for emphasis and focused measures.

Furthermore, governance has also been identified as relating to the implied and definite institutions and processes that frame power relations between actors and within the actions of actors.^57^ As power asymmetries between health providers (and communities) influence governance,^58^ more emphasis should be placed on the awareness of power relations between actors (such as governments and NGOs) and communities. Developing governance skills in front-line workers (HCWs acting at the community level) can promote awareness of power dynamics in managerial positions, promoting cooperation between health system actors.^59^ Given that HCWs are the predominant providers acting at the community level (the community–health system interface), a focus on the relational aspects of governance and leadership could help manage and right community–health system interface power imbalances.^16 60^

### Sociopolitical influences on governance and cholera reporting

When good governance is lacking or interfered with, through the effect of external influences, such as geopolitical factors and pressures (emphasised by the barrier ‘socioeconomic influences and politicisation of cholera responses’ from theme 4), reporting and containing cholera outbreaks are negatively influenced. These factors illustrate that health systems do not exist in isolation but within complex political, cultural, economic and ecological, systems on all levels, from local to international.^57^ External influences on governance functioning, such as disincentives to report cholera outbreaks, have been noted in many countries. In addition, the ongoing association cholera has with signs of inadequate modernity, that is, relating to a state of deficient WASH and infrastructural development, may mean that labelling a country as the place of origin of an outbreak may disincentivise reporting and hamper collaboration with local actors.^61 62^ Despite the broad term ‘cholera reporting country’ in use throughout this study highlighting the act of reporting cholera, employing it rather than terms that are income and lending based (and carrying historical/sociopolitical associations^63^), may avoid the notion that all countries with cholera are cholera endemic or impoverished (as both terms are negatively loaded).

Cholera is no longer reportable under the revised International Health Regulations. Still, denial of or downplaying cholera case numbers is a global health concern and a threat to surveillance efforts.^14 62 64^ Importantly, under-reporting provides incorrect governance information and can interfere with response planning and service delivery. Transparent data collection for disease reporting is necessary not only for global health security but also countries’ preparedness to manage outbreaks. Revision or supplementation of the IHR with reporting of subnational case numbers or other service delivery measurement data may be warranted to contain outbreaks.^65^ Importantly, revisions to the IHR and planned pandemic preparedness treaty need equity and cooperation as starting points.^66^

### Cooperation and the community

Cooperation is essential in facilitating cholera control, as noted in theme 2 relating to HSS, as well as in theme 8 concerning the community–health system interface. Cooperation’s importance is evident in humanitarian emergencies or fragile settings.^29 67^ Likewise, a recent review of cholera outbreaks in refugee settings highlighted coordination between the local Ministry of Health (government health provider) and external providers (NGOs), as enabling significant reductions in cholera spread, thereby directly benefiting most at-risk populations (refugees and internally displaced persons).^68^ The findings of theme 8 are mirrored in literature on the Ebola Virus Disease (EVD) epidemic in West Africa 2014–2016. Community leadership engagement and cooperation, enabled community mobilisation, case and contact tracing and surveillance in areas of distrust towards governmental institutions.^69 70^ Such cooperation is also essential in settings with cultural, linguistic and geographical barriers, such as those of nomadic pastoralists.^71^

### Trust

Notably, as found in theme 5 relating to community engagement and theme 9 with regard to the community–health system interface, trust is vital for community cooperation with infectious disease measures.^16^ Similarly, an extensive survey of Monrovian residents during the start of the EVD epidemic of 2014–2015 suggested that in fragile settings, trust in the government’s ability to manage the outbreak could be linked to adherence to, and agreement with, EVD prevention and control measures.^72^ Negative sociopolitical influences on trust,^73 74^ such as slow institutional responses to cholera outbreaks, can lead to a lack of community trust in government services and hence affect service utilisation at the community–health system interface.^75 76^ This is particularly true for marginalised communities who appear to trust less and have less power to act,^74^ and in fragile settings, such as those with a history of political instability and institutional neglect. To ameliorate these poor perceptions, government actors, if willing, could be particularly visible and supportive when introducing control measures. This would help build trust in governments that provide the needed services.^72^ In addition, opportunities for power transfers and collaboration at the community–health system interface could be created through initiatives such as participatory group model building; such a method of inviting exploration of the power dynamics between stakeholders can be especially helpful in fragile settings to promote successful interventions.^74^

### Stigma and cholera messaging

A community’s involvement in governance, and in turn collaboration and power sharing at the community–health system interface, is linked to that community’s trust in and sense of ownership in the health system.^77^ As noted in theme 6, stigma and marginalisation are potent barriers to participation by negating individual and community power, as noted in stigmatised communities in the Dominican Republic, Tanzania, Peru, the Democratic Republic of Congo and Brazil.^32 40 48 49 78^ Decontextualised language use and inappropriate communication strategies can reinforce this stigmatisation and ‘othering’ (marginalisation). Additionally, perceptions of community distrust towards government actors can be changed or reinforced.^62 75 76 79^ This effect was also noted in relation to EVD in West Africa in 2014–2016.^80^ Therefore, messaging in fragile settings needs to be carefully contextualised.^16 69^ Cholera messaging that inappropriately links personal attributes (such as personal hygiene), to cholera transmission routes (such as faecal-oral transmission routes) should be avoided, as this may lead to individual and community shame and marginalisation if illness is associated with hygiene.^48 75^

Of note, even though marginalised communities, or individuals, may be thought to need health initiatives, such as making long-term WASH changes, their ability to act on external initiatives (such as funding for latrine building), may be compromised by their disenfranchisement and lack of control of their greater circumstances. Poverty is a powerful deterrent to community engagement, and must be combatted alongside cholera interventions.^53 62 75 78^ Further and strikingly, national poverty (such as food insecurity) has recently been associated with an increased country cholera incidence rate.^81^ Failing to consider pre-existing inequalities, such as structural barriers (poor WASH or poverty) to cholera prevention and control measures, may lead to interventions increasing inequalities in certain communities.^82^

### Insecurity

Finally, insecurity, as identified in theme 1 relating to HSS, and theme 7 relating to the community–health system interface, is a crucial barrier to HSS, community empowerment and service delivery at the community–health system interface. Population displacement, due to people fleeing conflict areas, causes resource-poor refugee camp formation, where WASH and health service provision are often inadequate.^29 68 71 83 84^ Incomplete surveillance and disorganisation of health provision, increase cholera risk and harm service delivery, putting at-risk populations at a higher risk of cholera.^61 83^ Therefore, global solidarity and international pressure to end conflicts would benefit cholera prevention and control in settings made fragile by armed conflicts.

### Study limitations and strengths

The scoping review methodology employed in this study has some inherent limitations.^18^ As a quality appraisal is not usually included (and would have been difficult given the variety of included documents), any inherent weaknesses in the included documents may not be explicitly noted and may influence the analysis by misrepresenting an intervention or setting. However, an appraisal of the author and reviewer specified limitations of all included documents was performed (see online supplemental file 3 for an example) and considered during analysis. Furthermore, the selection of English language texts only, as well as the exclusion of documents focusing on knowledge, attitude and practice and WASH, are also noted as limitations, as is not including other sources of documents, such as grey literature. Additionally, a paradigm bias may exist, in that the literature may be biased towards studies that show positive results. Although optional, a consultative exercise with cholera experts may have helped to validate this study’s findings.

Notwithstanding, this study is strengthened by the transparent reporting in line with the PRISMA-ScR (see details in the checklist), a validated scoping review appraisal tool.^19 23^ Additionally, repeated iterative cycles of all stages of the methodological process and a large number of included documents allowed for a broad but detailed mapping of the selected evidence across multiple settings and periods. This increased validity and generalisability. Finally, this study’s conceptual exploration of the under researched areas of HSS, community engagement and especially the community–health system interface in cholera control, and subsequent new synthesis of available evidence regarding these, is a notable strength.

### Implications of findings for control, policy and research

This study identified and described three areas where facilitators of and barriers to community engagement and HSS overlapped; these were the themes describing factors at the community–health system interface. This space, where public service provision by health system actors (service delivery in terms of HS function) to community members occurs and where response processes occur (community engagement), is where sustained interventions for cholera control and response must have their effect. It is also where any interventions targeting HSS or community engagement are tested. We found that states of insecurity and community-driven violence acted as barriers at the interface. Cooperation and agreement between health providers and communities, when present, facilitated interactions, while lack thereof blocked these. Furthermore, poor governance practices, and the influence of greater contextual factors such as political and socioeconomic factors, influenced interactions and processes at the interface. Due to the crucial role that the community–health system interface plays in the sustained implementation of both HSS and community engagement interventions for cholera prevention and control, these findings appear to have substantial implications for the means to achieve the goals outlined in the GTFCC Roadmap 2030.^1^ Specifically, interventions focusing on the six described pillars, such as community engagement, WASH, OCV and HSS alone, may not be enough for sustained cholera control. Concerning HSS, the GTFCC Roadmap 2030 directs focus to, among others mentioned previously, effective management of supply chains in epidemic settings and improving staff capacity in endemic settings.^1^ However, good governance, in terms of political support, and collaboration and cooperation with community members and other health providers, is also critical to ensuring awareness and acceptance of HSS interventions in the health system, as well as in the community.

Regarding community engagement, the GTFCC Roadmap 2030 recommends increasing communication around WASH and safe hygiene practices and ‘mobilising community leaders as agents of change’.^1^ As noted in this study, increased communication alone may not enable safe WASH and hygiene practices as communities may, through greater social-political or economic factors, be unable to sustain such interventions.^73^ Finally, targeting ‘hotspots’ advocated by the GTFCC, which are likely to be in fragile settings, without considering existing power dynamics and trust or community confidence issues, may risk increasing distrust in government institutions. Community-driven violence could result, even when health services are provided by NGOs.^29^ Such targeting may even increase stigmatisation and marginalisation within communities^48 49^; this could also disempower communities. Given the importance of the community–health system interface, further research is needed to understand the context-specific determinants of communities’ trust in, and perceptions of state actors, as well as additional exploration of facilitating community empowerment drivers and mechanisms, especially in fragile settings. Likewise, evidence-based guidelines for ensuring sustained and pragmatic collaboration with communities are needed to eradicate cholera.

## Data Availability

Data are available on reasonable request.
