# Supplementary material for: Influences of community engagement and health system strengthening for cholera control in cholera reporting countries
Source: BMJ Glob Health. 2023 Dec 6;8(12):e013788. doi: 10.1136/bmjgh-2023-013788 (PMC10711916; doi:10.1136/bmjgh-2023-013788)
Supplement: Supplementary data [file bmjgh-2023-013788supp001.pdf]

Supplementary File 1. Full search terms used in electronic database searching

**PubMed**		
Cholera[mesh] OR cholera[Title/Abstract]	AND	Community participation[mesh] OR communit*[Title/Abstract] OR particip*[Title/Abstract] OR organi*[Title/Abstract] OR mobili*[Title/Abstract] OR engag*[Title/Abstract] OR involve*[Title/Abstract]
Cholera[mesh] OR cholera[Title/Abstract]	AND	((((health[Title/Abstract]) AND (((((((service[Title/Abstract]) OR (deliver*[Title/Abstract])) OR ("care work*"[Title/Abstract])) OR (work*[Title/Abstract])) OR (personnel*[Title/Abstract])) OR (organi*[Title/Abstract])) OR (admini*[Title/Abstract]))) OR ((((medical technolog*[Title/Abstract]) OR (information system*[Title/Abstract])) OR (govern*[Title/Abstract])) OR ((health[Title/Abstract]) AND ((financ*[Title/Abstract]) OR (mone*[Title/Abstract]))))) OR ((health) OR (healthcare))) OR (((((((Health care quality, access, and evaluation[mesh]) OR (health care facilities, manpower, and services[mesh])) OR (health services administration[mesh])) OR (Information systems[mesh])) OR (technology[mesh])) OR (healthcare financing[mesh])) OR (health care economics and organizations[mesh]))
**Web of Science**		
TS=cholera	AND	TS="community participation" OR (TS=(communit* OR particip* OR organi* OR mobili* OR engag* OR involve*)

TS=cholera	AND	((TS=health) OR (TS=healthcare)) OR ((TS=health) AND (TS=(service OR deliver* OR "care work*" OR work* OR personnel * OR organi* OR admini*))) OR (TS=("medical technolog*" OR "information system*" OR govern* OR "stakeholder involve*")) OR ((TS=health) AND (TS=(financ* OR mone*)))
**CINAHL**		
MH "Cholera" OR AB cholera	AND	(MH "Community Role") OR (MH "Community-Institutional Relations") OR (MH "Community Networks")) OR (communit* OR particip* OR organi* OR mobili* OR engag* OR involve*)
MH "Cholera" OR AB cholera	AND	health OR healthcare OR (health AND service OR deliver* OR "care work*" OR work* OR personnel* OR organi* OR admini*) OR medical technolog* OR "information system*" OR govern* OR "stakeholder involve*" OR (health AND financ* OR mone*) OR (MH "Stakeholder Participation")
