# Supplementary material for: Influences of community engagement and health system strengthening for cholera control in cholera reporting countries
Source: BMJ Glob Health. 2023 Dec 6;8(12):e013788. doi: 10.1136/bmjgh-2023-013788 (PMC10711916; doi:10.1136/bmjgh-2023-013788)
Supplement: Supplementary data [file bmjgh-2023-013788supp002.pdf]

Supplementary File 2. Example data extraction sheet for three selected documents

Identifiers				*Description*						*Methodology*							
Title	Author(s)	Author affiliation	Year	Country	Setting	Context		Purpose of study	Dates of study	Study design	Key population	Data collection	Limitations		Community engagement		
						cholera	situational						Author identified	Reviewer identified	*Facilitator*	*Barrier*	*Mechanism*
A rapid assessment of the implementation of integrated disease surveillance and response system in Northeast Nigeria, 2017	Ibrahim LM, Stephen M, Okudo I, Kitgakka SM, Mamadu IN, Njai IF et al.	multinational NGO	2020	Nigeria	rural; multiple regions	endemic	armed conflict	determine status of implementation of surveillance system in chosen states		mixed methods	surveillance officers	n= 34 surveys	selection bias; information bias	respondent bias; social desirability bias			
Community led total sanitation for community based disaster risk reduction: A case for non-input humanitarian relief.	Mlenga DH, Baraki YA	academic	2016	Swaziland	rural; multiple regions	endemic		determine effect of community led total sanitation project or subsidy based sanitation intervention on sanitation coverage in chosen communities	February 2013-June 2014	experimental mixed methods	rural community members	n= 200 households		information bias; confirmation bias; selection bias; respondent bias; social desirability bias; recall bias; confounding	traditional leadership engagement		mentoring and encouragement for ongoing community activity
Medication supply chain management through implementation of a hospital pharmacy computerized inventory program in Haiti	Holm MR, Rudis MI, Wilson JW	academic	2015	Haiti	urban setting	outbreak	earthquake	develop and implement electronic pharmacy supply chain management system	March 2011-February 2012	quasi experimental case study with implementation research	nurses, pharmacists in hospital	n= 75 users		confounding; respondent bias; social desirability bias; confirmation bias			

Identifiers				***Description***						***Methodology***					**Community engagement**		
**Title**	**Author(s)**	**Author affiliation**	**Year**	**Country**	**Setting**	**Context**		**Purpose of study**	**Dates of study**	**Study design**	**Key population**	**Data collection**	**Limitations**				
						cholera	situational						Author identified	Reviewer identified	*Facilitator*	*Barrier*	*Mechanism*
A rapid assessment of the implementation of integrated disease surveillance and response system in Northeast Nigeria, 2017	Ibrahim LM, Stephen M, Okudo I, Kitgakka SM, Mamadu IN, Njai IF et al.	multinational NGO	2020	Nigeria	rural; multiple regions	endemic	armed conflict	determine status of implementation of surveillance system in chosen states		mixed methods	surveillance officers	n= 34 surveys	selection bias; information bias	respondent bias; social desirability bias			
Community led total sanitation for community based disaster risk reduction: A case for non-input humanitarian relief.	Mlenga DH, Baraki YA	academic	2016	Swaziland	rural; multiple regions	endemic		determine effect of community led total sanitation project or subsidy based sanitation intervention on sanitation coverage in chosen communities	February 2013-June 2014	experimental mixed methods	rural community members	n= 200 households		information bias; confirmation bias; selection bias; respondent bias; social desirability bias; recall bias; confounding	traditional leadership engagement		mentoring and encouragement for ongoing community activity
Medication supply chain management through implementation of a hospital pharmacy computerized inventory program in Haiti	Holm MR, Rudis MI, Wilson JW	academic	2015	Haiti	urban setting	outbreak	earthquake	develop and implement electronic pharmacy supply chain management system	March 2011-February 2012	quasi experimental case study with implementation research	nurses, pharmacists in hospital	n= 75 users		confounding; respondent bias; social desirability bias; confirmation bias			

Community and Health system interaction		Interesting quote	Conclusion	
Description	Mechanism		Author	Reviewer
		of motorbikes. This ban has negatively impacted the retrieval of IDSR data and the ability to perform supportive supervision."; "It is obvious that paper-based reporting leads to serious limitations in the transmission of the data from the point of generation to the higher level (LGA)"	surveillance system deficiencies in staff capacity and supervision	armed conflict and poor oversight impeding health system functioning
generation of suspicion and poor social cohesion resulting in poor mobilisation	different motivational methods for community led interventions used in same community (subsidy versus non-subsidy)	"When asked how and why it was possible for others in the same community, who had not received incentives, to construct latrines, 42% of the respondents highlighted that, the members who were able to construct latrines had received incentives secretly."	subsidising sanitation projects can lead to dependency on assistance	uniformity in community approaches for sanitation key to acceptance
		"...our Mayo Clinic staff were physically present during the initial 'hands-on' implementation and teaching of PCIP, during the rapid PDSA cycling system improvement process, as well as during subsequent trips to 'reinforce' the education."	simple, multilingual, web-based program implementable in low resource setting	supportive supervision and context specific adjustments key to implementation of information system strengthening
