# Supplementary material for: Influences of community engagement and health system strengthening for cholera control in cholera reporting countries
Source: BMJ Glob Health. 2023 Dec 6;8(12):e013788. doi: 10.1136/bmjgh-2023-013788 (PMC10711916; doi:10.1136/bmjgh-2023-013788)
Supplement: Supplementary data [file bmjgh-2023-013788supp003.pdf]

BMJ Publishing Group Limited (BMJ) disclaims all liability and responsibility arising from any reliance placed on this supplemental material which has been supplied by the author(s)

Supplementary File 3. Example of coding process used in thematic analysis

**Text**	**Code**		**Theme**
"Community-based involvement in outbreak response is critical in regions often troubled by years of civil conflict and in populations historically suspicious of outsiders or government involvement." *(22)*	Community representatives' involvement enables trust	Facilitator: Community representatives	Trust building in Community-Health system
"Meeting with community leaders before and during the vaccination campaign was important to counter doubts voiced in some popular media on the efficacy and potential harmful effect of OCV." *(15)*	Trust and transparency through community representatives' communication	Facilitator: Community representatives	
"...the Caliph himself lobbied for the water to be chlorinated and publicly broke his own basin as an example to others back in 2005 – not the first time the Marabouts had taken a lead in matters of hygiene." *(23)*	Trust through religious leadership assists in communication strategy	Facilitator: Community representatives	
