# Supplementary material for: Influences of community engagement and health system strengthening for cholera control in cholera reporting countries
Source: BMJ Glob Health. 2023 Dec 6;8(12):e013788. doi: 10.1136/bmjgh-2023-013788 (PMC10711916; doi:10.1136/bmjgh-2023-013788)
Supplement: Supplementary data [file bmjgh-2023-013788supp004.pdf]

Supplementary file 4. Number of search results by electronic database searched

**Cholera and 'Community Engagement'**	**Number of results**
PubMed	3193
Web of Science	4867
CINAHL	406
**Cholera and 'Healthcare System Strengthening'**	
PubMed	6101
Web of Science	2195
CINAHL	756
